# The incidence and mechanism of sunitinib-induced thyroid atrophy in patients with metastatic renal cell carcinoma

**DOI:** 10.1038/sj.bjc.6606029

**Published:** 2010-12-14

**Authors:** N Shinohara, M Takahashi, T Kamishima, H Ikushima, N Otsuka, A Ishizu, C Shimizu, H Kanayama, K Nonomura

**Affiliations:** 1 Department of Renal and Genitourinary Surgery, Hokkaido University Graduate School of Medicine, North-15, West-7, Kitaku, Sapporo 060-8638, Japan; 2 Department of Urology, University of Tokushima Graduate School, Kuramae-cho 3-choume, Tokushima 770-8503, Japan; 3 Department of Radiology, Hokkaido University Graduate School of Medicine, North-15, West-7, Kitaku, Sapporo 060-8638, Japan; 4 Department of Radiology, University of Tokushima Graduate School, Tokushima, Kuramae-cho 3-choume, Tokushima 770-8503, Japan; 5 Department of Pathology, Hokkaido University Graduate School of Medicine, North-15, West-7, Kitaku, Sapporo 060-8638, Japan; 6 Faculty of Health Sciences, Hokkaido University, North-12, West-5, Kitaku, Sapporo 060-0812, Japan; 7 Division of Laboratory and Transfusion Medicine, Hokkaido Uinversity Hospital, North-14, West-5, Kitaku, Sapporo 060-8648, Japan

**Keywords:** thyroid atrophy, hypothyroidism, sunitinib, renal cell carcinoma

## Abstract

**Background:**

To elucidate the incidence and mechanisms of sunitinib-induced thyroid atrophy, we investigated serial volumetric and functional changes, and evaluated histological changes of the thyroid gland in metastatic renal cell carcinoma patients who received sunitinib.

**Methods:**

Thyroid volume (by computed tomography volumetry) and thyroid function were measured at baseline, during the treatment, and at post-treatment periods. Histological evaluation of the thyroid gland was performed in four autopsied patients.

**Results:**

The median reduction rate in thyroid volume at last evaluation during sunitinib treatment was 30% in all 17 patients. The incidence of hypothyroidism during sunitinib treatment was significantly higher in the high reduction rate group (*n*=8; more than 50% reduction in volume) than in the low reduction rate group (*n*=9; less than 50% reduction in volume). Half of the patients in the high reduction rate group exhibited a transient thyroid-stimulating hormone suppression, suggesting thyrotoxicosis during sunitinib treatment. Histological evaluation demonstrated atrophy of thyroid follicles and degeneration of follicular epithelial cells without critical diminution of vascular volume in the thyroid gland.

**Conclusion:**

Thyroid atrophy is frequently observed following sunitinib treatment and may be brought about by sunitinib-induced thyrotoxicosis or the direct effects of sunitinib that lead to degeneration of thyroid follicular cells.

Sunitinib malate (SUTENT, Pfizer Inc., New York, NY, USA) is an oral, multitargeted tyrosine kinase inhibitor of vascular endothelial growth factor receptors, platelet-derived growth factor receptors, stem cell factor receptor (c-KIT), and is rearranged during transfection. It has been approved for the treatment of gastrointestinal stromal tumour and metastatic renal cell carcinoma (RCC). Single-agent sunitinib showed unprecedented antitumour activity in two phase II trials of patients with metastatic RCC, demonstrating an objective response rate of 33% (Motzer *et al*, 2006a, 2006b). Furthermore, sunitinib demonstrated superior first-line efficacy over IFN-α, with significantly greater progression-free survival (PFS) (Motzer *et al*, 2007, 2009). On the basis of these results, sunitinib is one of the standard drugs for treatment-naïve RCC in the National Comprehensive Cancer Network treatment guidelines.

Although sunitinib is a potent drug, it is relatively toxic and is reported to have several treatment-related adverse events, such as myelosuppression, hypertension, hand-foot syndrome, and diarrhoea (Motzer *et al*, 2009; Theou-Anton *et al*, 2009). Of these, hypothyroidism has recently been recognised as one of the most frequently observed adverse events and a clinically relevant toxicity of sunitinib. In a prospective study by Desai *et al*, hypothyroidism developed in 15 (36%) of 42 patients with gastrointestinal stromal tumour treated with sunitinib for a mean of 50 weeks (Desai *et al*, 2006). Rini *et al* reported that hypothyroidism occurred in 56 (85%) of 66 patients with metastatic RCC at a median of two cycles of sunitinib treatment (Rini *et al*, 2007). Although the mechanism behind this complication remains unclear, it is considered that treatment with levothyroxine sodium can control subclinical and overt hypothyroidism induced by sunitinib.

Sunitinib is reported to induce another type of toxicity of the thyroid, thyroid atrophy, in some patients. In the study by Desai *et al*, severe thyroid atrophy occurred in two patients who experienced sunitinib-induced thyrotoxicosis, which was probably due to destructive thyroiditis (Desai *et al*, 2006). On the other hand, Mannavola *et al* reported that, in 11 patients with gastrointestinal stromal tumour, whose thyroids were evaluated by ultrasonography, no changes in volume and/or echographic pattern were recorded during sunitinib treatment (Mannavola *et al*, 2007). Thus, no consensus has yet been reached on whether sunitinib induces thyroid atrophy during the treatment. Furthermore, there are no data on histological changes in the thyroid gland in patients who received sunitinib. To elucidate the incidence and the underlying mechanisms of thyroid atrophy, we investigated the serial volumetric and functional changes and evaluated histological changes of the thyroid gland in patients with metastatic RCC who received sunitinib.

## Materials and Methods

### Patients

A total of 17 patients with metastatic RCC were enrolled in a prospective observational study. In total, 12 patients were treated in a phase II trial investigating single-agent sunitinib therapy in Japanese patients with metastatic RCC (Uemura *et al*, 2010). The remaining five patients were enrolled after April 2008 as a cohort of a post-marketing surveillance study. Patients having a history of medical treatment for thyroid disease and those who underwent sunitinib therapy for less than 4 weeks were excluded from the present study.

In most patients, sunitinib was administered at a starting dose of 50 mg orally, once daily, in the morning, without regard to meals, in repeated 6-week cycles according to Schedule 4 out of 2 (4 weeks on treatment, followed by 2 weeks off). Patients were monitored for toxicity, and doses were adjusted following individual patient tolerance according to the following protocol. Doses were reduced to 37.5 mg per day in cases of treatment-related grade >3 adverse events, and additionally to a minimum dose of 25 mg per day if toxicities persisted. Treatment was continued until one of the following occurred: disease progression; requirement for additional anticancer treatment; development of left ventricular dysfunction; or treatment withdrawal.

Tumour assessments were based on the Response Evaluation Criteria in Solid Tumours (Therasse *et al*, 2000), with computed tomography (CT) obtained before treatment and on day 1 of treatment cycles. All patients gave written informed consent. The present study was approved by the institutional review board at two institutions and was conducted in accordance with the International Conference on Harmonization Guidelines for Good Clinical Practice.

### Evaluation of thyroid function

The thyroid function test (TFT) was assessed at baseline and on day 1 of each treatment cycle in all but one patient, for whom the collection of a blood sample at baseline was not carried out. As a TFT, we measured serum TSH (thyroid-stimulating hormone; reference range 0.38–4.31 mIU l^–1^), free triiodothyronine (reference range 2.1–3.8 pg ml^–1^), and free thyroxine ( reference range 0.82–1.63 ng per 100 ml). A TFT was always carried out before performing a CT scan with iodium contrast. In some patients, antithyroglobulin antibody (reference range 0–13.6 IU ml^–1^) and antithyroid peroxidase antibody (reference range 0–3.2 IU ml^–1^) were assessed.

Subclinical hypothyroidism is defined as serum TSH above the upper limit of normal values, with fT4 within normal limits at baseline and on day 1 of each cycle. Overt hypothyroidism is defined as low serum fT4, together with elevated TSH.

Thyroid hormone replacement therapy using levothyroxine sodium was initiated in patients with at least two consecutive serum TSH measurements >10 mIU l^–1^ or with any symptoms compatible with hypothyroidism according to the recommendation in Desai *et al* (2006).

### Measurement of thyroid gland volume with CT scan

**Figure 1:**
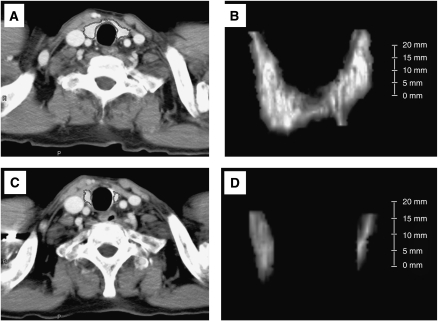
Measurement of thyroid gland volume using CT scan. A cross-sectional image (**A**) and three-dimensional (3D) reconstruction image (**B**) of the thyroid gland in CT scan with contrast at baseline in patient 5 (thyroid volume 7.8 ml). A cross-sectional image (**C**) and 3D reconstruction image (**D**) of the thyroid gland in the same patient 15 months after the start of sunitinib treatment (thyroid volume was 1.2 ml).

In all patients, thyroid volume was measured serially by CT volumetry on a cervical-pelvic CT scan, which was evaluated every 1 or 2 cycles to assess tumour response. CT volumetry was performed by two radiologists (TK and HI) who had no clinical information on thyroid function, using a commercially available workstation (ZIOSOFT, ZIOSOFT Inc., Tokyo, Japan) (Kato *et al*, 2009). With ZIOSOFT, three-dimensional reconstruction images of thyroid glands were created using contrast-enhanced CT images of the renal parenchymal phase with a slice thickness of 5 mm (Figure 1A). After creating three-dimensional reconstruction images of thyroid glands (Figure 1B), the volume of thyroid glands was obtained. By CT volumetry, the volume of thyroid gland in patient 5 (before sunitinib treatment) was measured to be 7.8 ml. Figures 1C and D showed a cross-sectional image and a three-dimensional reconstruction image of the thyroid gland, respectively, 15 months after the start of sunitinib treatment in the same patient (thyroid volume=1.2 ml).

### Histological evaluation of thyroid gland at autopsy

Histological evaluation of the thyroid gland was performed in the four patients who received sunitinib and subsequently died of progressive disease, and in a 57-year-old male patient who died of cardiac attack. The patient who died of cardiac attack was never administered sunitinib. Two pathologists (NO and AI), who had no clinical information on thyroid function, evaluated the haematoxylin–eosin (HE)-stained specimens of the thyroid glands obtained from the four autopsied patients. Furthermore, immunohistochemistry with antibody to CD34 was undertaken to analyse thyroidal capillarisation and arterialisations.

### Statistical analysis

Groups of subjects were compared using the Mann–Whitney *U-*test (continuous variables) or using Pearson's *χ*^2^-test or Fischer's exact test (discrete variables). The PFS was defined as the interval from the start of sunitinib treatment until confirmation of disease progression or death. The PFS was evaluated by the Kaplan–Meier method with significance determined using the log-rank test. For all statistical analyses, *P*<0.05 was regarded as statistically significant.

## Results

The backgrounds of the 17 patients are summarised in Table 1. The median age was 62 years (range 23–78). Nine patients (53%) had undergone previous treatment with IFN-*α*. At the time of final analysis, patients had received a median of six cycles of sunitinib treatment (range 1–23). The objective response rate by Response Evaluation Criteria in Solid Tumours was 53%, with one (6%) and eight patients (47%) achieving confirmed complete response and confirmed partial response, respectively. Stable disease for 6 months or longer was also obtained in three patients (18%). Median PFS of all 17 patients was 11.9 months. In terms of the TFT at baseline, 12 patients had no abnormal TFT and four patients showed subclinical hypothyroidism. The median serum TSH concentration at baseline was 1.63 mIU l^–1^ (range 0.53–10.14). There was no significant difference in serum TSH concentration (mean+s.d.) at baseline between patients who received IFN-*α* and those who did not (1.69+0.87 *vs* 5.22+4.19, *P*=0.244). Thyroid autoantibodies, antithyroglobulin and antithyroid peroxidase antibodies were not detected in any of the six patients assessed, of whom two patients had subclinical hypothyroidism and the others showed euthyroid at baseline. No patient had any symptoms suggesting hypothyroidism at entry.

With regard to thyroid function impairment during treatment with sunitinib, nine patients (53%) developed hypothyroidism and all but one patient (patient 8) received an appropriate dose of levothyroxine sodium (50–150 *μ*g per day). Of them, four patients experienced a transient TSH suppression suggesting thyrotoxicosis before the development of overt hypothyroidism. The remaining eight patients retained euthyroid status throughout treatment with sunitinib, with a median of two cycles (range 1–6). All four patients with subclinical hypothyroidism at baseline developed overt hypothyroidism during sunitinib treatment. In the 12 patients with euthyroid at baseline, hypothyroidism occurred in four patients and eight patients retained their euthyroid status. These results suggest that hypothyroidism is likely to occur in patients with subclinical hypothyroidism at baseline, than in those with euthyroid (*P*=0.083).

**Figure 2:**
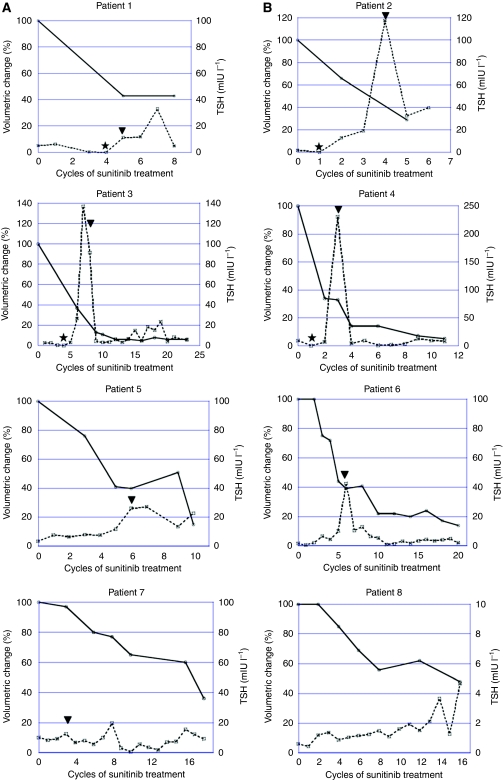
Serial changes in thyroid volume and serum TSH level in the high reduction rate group (*n*=8). The four patients experienced a transient TSH suppression suggesting thyrotoxicosis before the development of hypothyroidism (**A**). A maximum suppression level of serum TSH and duration of TSH suppression were 0.03 mIU l^–1^ and 28 days, 0.05 mIU l^–1^ and 14 days, 0.08 mIU l^–1^ and 28 days, and 0.03 mIU l^–1^ and 14 days, in patients 1, 2, 3, and 4, respectively. The remaining four patients did not show signs of thyrotoxicosis (**B**). Bold lines delineate the change of thyroid gland volume during sunitinib treatment and dotted lines demonstrate serial change of serum TSH level measured at day 1 of each cycle. ★, the development of thyrotoxicosis. ▾, the start of levothyroxine. TSH, thyroid-stimulating hormone.

**Figure 3:**
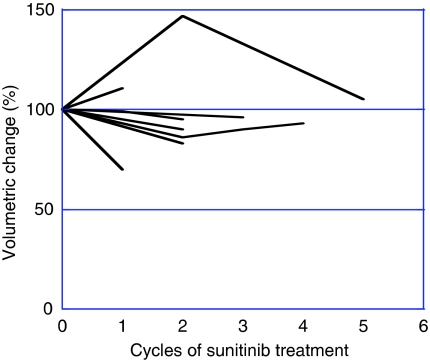
Serial changes in thyroid volume in the low reduction rate group (*n*=9).

In 17 patients evaluated in the present study, the median thyroid volume at baseline was 13.6 ml (range 5.8–20.2 ml). There was no difference in thyroid volume (mean+s.d.) at baseline between patients who received IFN-*α* previously and those who did not (13.99+5.39 *vs* 13.50+4.17 ml, *P*=0.810). As dispersion of thyroid volume at baseline was shown among the patients, the changes in thyroid volume were evaluated as the rate of reduction in thyroid volume at each measurement during treatment with sunitinib relative to that at baseline. The median reduction rate was 30% at the last evaluation during sunitinib treatment (median six cycles, range 1–23 cycles). On the basis of the reduction rate in thyroid volume, we could clearly stratify the patients into two groups: the high reduction rate group (more than 50% reduction; eight patients) and the low reduction rate group (less than 50% reduction; nine patients). The median reduction rate of thyroid volume and median thyroid volume at the last evaluation during sunitinib treatment were 78% (range 52–95) and 2.1 ml (range 0.4–8.0), respectively, in the high reduction rate group. The serial changes of thyroid volume and serum TSH level in each patient of the high reduction rate group are shown in Figures 2A and B. The median reduction rate of thyroid volume and median thyroid volume at the last evaluation during sunitinib treatment were 10% (range 0–30) and 13.0 ml (range 2.8–20.9), respectively, in the low reduction rate group (Figure 3). Patient characteristics of the two groups are listed in Table 2. The patients of the high reduction rate group received significantly more cycles of sunitinib treatment and had a significantly higher response rate than the patients of the low reduction rate group. Furthermore, median PFS was significantly longer in the former than in the latter. Although there was no significant difference in the incidence of subclinical hypothyroidism at baseline between the two groups, the incidence of hypothyroidism during sunitinib treatment was significantly higher in the high reduction rate group (100%) than in the low reduction rate group (11%). In addition, four of eight patients in the high reduction rate group exhibited signs of thyrotoxicosis during sunitinib treatment. The maximum TSH value during sunitinib treatment was also significantly higher in the high reduction rate group.

Of 17 patients evaluated in the present study, six have continued to receive sunitinib treatment, whereas sunitinib was stopped in 11 patients because of disease progression. In the latter group, four patients died of cancer in the period after the discontinuation of sunitinib therapy. We evaluated the changes in TSH value and thyroid volume in four of the remaining seven patients who underwent subsequent therapy. No increase in thyroid volume was observed in two patients of the high reduction rate group during sunitinib treatment. These two patients experienced overt hypothyroidism with a profound elevation in TSH (326 mIU l^–1^, 123 mIU l^–1^) after the discontinuation of sunitinib and levothyroxine sodium, in accordance with the suggestion made by Mannavola *et al* (2007). On the other hand, changes in thyroid volume and TSH value did not occur in the two patients belonging to the low reduction rate group.

**Figure 4:**
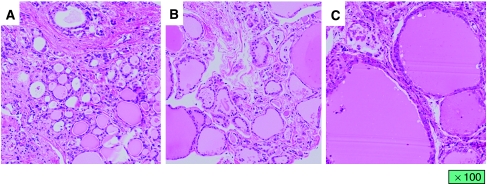
Histological changes in the thyroid gland. (**A**) The thyroid gland obtained from the patient who received sunitinib for 15 months (patient 5). The patient underwent best supportive care for 6 months after the discontinuation of sunitinib therapy and no change in thyroid gland volume was observed. (**B**) The thyroid gland obtained from the patient who received sunitinib for 2 months (patient 12). The patient died of cancer 1 month after discontinuation of sunitinib therapy. (**C**) The thyroid gland obtained from the patient who died of cardiac attack without a past history of thyroidal disease.

**Figure 5:**
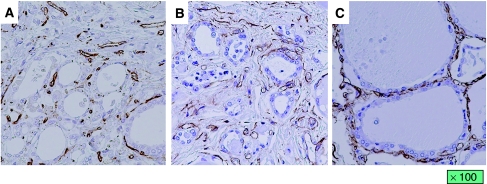
Vascular distribution of the thyroid gland. (**A**) The thyroid gland obtained from the patient who received sunitinib for 15 months (patient 5). (**B**) The thyroid gland obtained from the patient who received sunitinib for 2 months (patient 12). (**C**) The thyroid gland obtained from the patient who died of cardiac attack.

We also evaluated histological changes in the thyroid gland in the four autopsied patients. Although atrophy of thyroid follicles and degeneration of follicular epithelial cells were observed in all four patients, two patients (cases 2 and 5) of the high reduction rate group who received sunitinib for a long period had more marked changes in the thyroid gland than the two patients (cases 12 and 15) who underwent short-term treatment of sunitinib who belonged to the low reduction rate group (Figure 4). In the thyroid glands without atrophy, vessels were distributed around the follicles. On the other hand, the mesh distribution of vessels was destroyed with destruction of follicles in the two patients who belonged to the high reduction rate group. However, the volume of vessels in the thyroid gland was relatively well preserved even in the two patients with marked thyroid atrophy (Figure 5).

## Discussion

The present study revealed that sunitinib can induce a reduction in thyroid volume, thyroid atrophy, as well as hypothyroidism. It is well known that hypothyroidism is frequently associated with sunitinib treatment (Desai *et al*, 2006; Mannavola *et al*, 2007; Rini *et al*, 2007; Wolter *et al*, 2008). In the present study, about half of the patients evaluated developed hypothyroidism after a median of two cycles of treatment. Sunitinib-induced hypothyroidism is common in Japanese and Caucasian patients. On the other hand, whether thyroid atrophy is caused by sunitinib treatment has remained unclear. To clarify the actual effect of sunitinib on the thyroid gland, we measured serial changes in thyroid volume and evaluated histological changes in the thyroid gland in patients who received sunitinib.

Although the size of the thyroid gland is generally evaluated by ultrasonography, we measured it by CT volumetry on cervical-pelvic CT scans. Although there is the problem of radiation exposure in CT volumetry, unlike in ultrasonographic evaluation, operator-dependent bias for the measurement of thyroid volume may be small in CT volumetry (Nygaard *et al*, 2002). The median thyroid volume measured by CT volumetry in the 17 patients before sunitinib treatment was 13.6 ml. Furthermore, thyroid volume at baseline was not affected by previous IFN-*α* treatment. Lee *et al* recorded a thyroid volume of 17.5+6.6 ml by CT measurement in their study (Lee *et al*, 2006). The standard volume of thyroid gland in adults has been reported as 13–18 ml, and the baseline volume in our patients could be considered as equivalent to this adult standard (Langer, 1989).

In patients receiving sunitinib, a serial reduction in thyroid volume occurred in 8 of the 17 patients evaluated. There have been a few studies on changes in thyroid size in patients receiving sunitinib treatment. Desai *et al* reported that two patients with destructive thyroiditis had marked thyroid atrophy that caused hypothyroidism (Desai *et al*, 2006). More recently, Rogiers *et al* reported that marked shrinkage of thyroid volume during treatment with sunitinib was observed in two RCC patients with a preexisting nodular thyroid gland (Rogiers *et al*, 2010). On the other hand, Mannavola *et al* reported that there was no evidence of thyroid atrophy in patients undergoing sunitinib treatment (Mannavola *et al*, 2007). It is questionable whether their observation period was long enough, as our study showed that a reduction in thyroid volume of more than 50% occurred in most patients who received more than six cycles of treatment. All the eight patients with more than 50% reduction in thyroid size had hypothyroidism, and half of them developed it after a transient TSH suppression, suggesting thyrotoxicosis. From the data on thyroid autoantibodies, a transient TSH suppression in two patients does not appear to result from autoimmune-mediated hyperthyroidism. Furthermore, we have to consider the possibility that thyrotoxicosis might be brought about by the iodine contrast medium that was used during the CT scan. However, the effect of the iodine contrast medium on the development of thyrotoxicosis does not appear to be too strong because a TFT was carried out before performing a CT scan in all patients. These results suggest that thyrotoxicosis, which was induced by sunitinib itself, would be one of the mechanisms that result in thyroid atrophy.

To clarify the effects of sunitinib on the thyroid gland and the mechanism underlying thyroid atrophy, a histological evaluation of the thyroid gland was carried out in patients who underwent autopsy. To the best of our knowledge, histological examination of the thyroid gland has not been reported in patients who received sunitinib. We found that there was less damage to the blood supply of the thyroid gland, but there were atrophic thyroid follicles and degeneration of follicular epithelial cells. These changes were pronounced in patients with severe thyroid atrophy after long-term sunitinib treatment. The patients who underwent received short-term treatment without changes in thyroid volume also showed atrophic thyroid follicles and degeneration of follicular epithelial cells. Kamba *et al* reported that a loss of thyroid homeostasis was associated with inhibition of vascularisation by a tyrosine kinase inhibitor in a mouse model and that this might be one of the causes of hypothyroidism and thyroid atrophy (Kamba *et al*, 2006). Makita *et al* described the RCC patient who displayed overt hypothyroidism with an atrophic thyroid during on-periods in the sunitinib treatment cycles and who showed a recovery of thyroid size during off-periods (Makita *et al*, 2010). They speculated that thyroid function and volume depended on vascularity, which was negatively regulated by sunitinib. On the other hand, Mannavola *et al* demonstrated that there was no reduction in thyroid blood flow in patients receiving sunitinib as shown in ultrasonographic evaluation with colour Doppler, a finding that supports our results (Mannavola *et al*, 2007). Salem *et al* showed that sunitinib functions on the thyroid gland by inhibiting the growth of FRTL-5 thyroid cells in a time-related and dose-related manner (Salem *et al*, 2008). The degeneration of thyroid follicular cells revealed by our study may indicate a direct effect of sunitinib on these cells.

We also examined whether thyroid volume increased after discontinuation of sunitinib. However, there were no patients who showed an increase in thyroid volume after discontinuation. Furthermore, two patients developed a marked elevation in serum TSH and symptoms of hypothyroidism after discontinuation of both sunitinib and levothyroxine sodium. Mannavola *et al* claimed that patients with hypothyroidism caused by sunitinib treatment would improve spontaneously after cessation of treatment with sunitinib and levothyroxine sodium (Mannavola *et al*, 2007). However, our results suggest that hypothyroidism associated with severe thyroid atrophy caused by sunitinib may be irreversible, at least during a short-term follow-up.

The present study demonstrated that sunitinib causes not only hypothyroidism but also thyroid atrophy in RCC patients who receive the drug over a long period, and that this can result in irreversible hypothyroidism. Possible causes for this are considered to be sunitinib-induced thyrotoxicosis in some patients and a direct effect of sunitinib that leads to degeneration of thyroid follicular cells in others.

## Figures and Tables

**Table 1:** Baseline demographic and clinical characteristics of 17 patients

					**Thyroid autoantibody**		**At baseline**			**During sunitinib-Tx**		
**Patient**	**Gender/age (year)**	**Pre-Tx with IFN/duration (months)**	**Total cycle of sunitinib**	**Best response by sunitinib**	**Thyroglobilin antibody (IU** **ml^–1^)**	**TPO antibody (IU** **ml^–1^)**	**Thyroid volume (ml)**	**TSH (mIu** **l^–1^)**	**Status**	**Thyroid volume (ml)**	**TSH (mIu** **l^–1^)**	**Status**
1	F/44	No	9	PR	<0.15	<0.15	18.5	5.06	Hypo	8	33.15	Hypo^a^
2	F/62	No	6	PR	<0.15	<0.15	8.3	1.6	Eu	2.4	117.53	Hypo^a^
3	M/65	No	23	CR	—	—	17.3		Unknown	1.1	137.13	Hypo^a^
4	M/64	No	11	SD	—	—	10.6	9.43	Hypo	0.4	230.89	Hypo^a^
5	M/69	Yes/6	10	PR	<0.15	<0.15	7.8	3.62	Eu	1.2	27.23	Hypo
6	F/56	Yes/3	20	PR	—	—	11.6	1.86	Eu	1.7	42.53	Hypo
7	F/59	No	18	PR	—	—	18.2	10.14	Hypo	6.6	19.8	Hypo
8	F/66	No	16	PR			14.3	0.61	Eu	6.8	4.69	Hypo
9	M/52	Yes/6	6	PR	—	—	21	1.29	Eu	22	3.06	Eu
10	F/23	No	2	PD	<0.15	<0.15	8.8	8.65	Hypo	7.9	12.72	Hypo
11	M/65	Yes/24	3	PR	<0.12	<0.05	13.6	1.66	Eu	13	3.71	Eu
12	M/55	Yes/3	2	PD	—	—	11.4	0.84	Eu	9.5	3.91	Eu
13	M/64	No	2	PD	—	—	12	1.07	Eu	10	3.93	Eu
14	M/78	Yes/6	2	SD	—	—	20.2	2.24	Eu	19.2	2.3	Eu
15	F/59	Yes/6	1	PD	<0.12	<0.05	5.8	1.49	Eu	2.8	3.14	Eu
16	M/76	Yes/20	4	SD	—	—	15.6	0.65	Eu	14	3.51	Eu
17	M/45	Yes/2	1	PD	—	—	18.9	1.57	Eu	20.9	1.73	Eu

Abbreviations: CR=complete response; Eu=euthyroid; Hypo=hypothyroidism; PD=progressive disease; PR=partial response; SD=stable disease; TPO=thyroid peroxidase; TSH=thyroid stimulating hormone; Tx=treatment.

a Evidence of thyrotoxicosis before experiencing hypothyroidism.

**Table 2:** Patient characteristics upon stratification into two groups by the rate of reduction in thyroid volume

	**High reduction rate group**	**Low reduction rate group**	***P*-value**
*Gender*			
Female/male	5/3	2/7	0.234
			
*Age, years*			
Median (range)	63 (44–69)	59 (23–78)	0.797
			
*Sunitinib Tx, cycles*			
Median (range)	13.5 (6–23)	2 (1–6)	0.001
			
*ORR*			
CR/PR	7	2	
SD	1	2	
PD	0	5	0.012
50% PFS, months	Not reach(⩾14.9)	2.3	0.004
			
*Thyroid status at baseline*			
Hypothyroidism	3	1	
Euthyroid	4	8	
Unknown	1	0	0.243
			
*TSH at baseline, mIU* *l*^*–1*^			
Median (range)	3.62 (0.61–10.14)	1.57 (0.52–8.65)	0.314
			
*Thyroid volume at baseline, ml*			
Median (range)	13.0 (7.8–18.5)	13.6 (5.8–21.0)	0.797
			
*Thyroid status during*			
Sunitinib-Tx			
Hypothyroidism	8^a^	1	
Euthyroid	0	8	0.001
			
*Maximum TSH during*			
Sunitinib-Tx, mIU l^–1^			
Median (range)	37.84 (4.69–230.89)	3.51 (1.73–12.72)	0.001
			
*Supplement of L-T4*			
During sunitinib-Tx	7	1	0.008

Abbreviations: CR=complete response; ORR=objective response rate; PD=progressive disease; PFS=progression-free survival; PR=partial response; SD=stable disease; TSH=thyroid stimulating hormone; Tx=therapy.

a Four patients experienced hyperthyroidism before hypothyroidism.
